# Support Your Home Journals

**Published:** 1887-02

**Authors:** 


### SUPPORT YOUR HOME JOURNALS.

Here in the South our physicians should bear in mind that they owe a duty to their own section, in the matter of medical literature. They should encourage and support their home journals. They should not only subscribe for them but contribute to their pages. The South is not wanting in medical knowledge and talent, and if they have any pride in the medical progress of our section they should make known through their home journals all that they have gleaned in the fields of study and experience.

We appeal to our patrons to do their duty toward the RECORD in this respect; we would have them feel and take an interest in it as their own organ, and as a representative of the physicians in their own section. We ask your aid and influence in extending our list of subscribers, and in co-operating with us in our efforts to benefit the profession and to promote the progress of medical science in the Southern States.

